# Assessing the Influence of Innovation Characteristics on the Implementation Process of an Optimised Tuberculosis Diabetes Integrated Care Package (Opt TBD) -A Mixed Method Study Protocol

**DOI:** 10.12688/f1000research.165171.1

**Published:** 2025-06-18

**Authors:** Fatima Khalid, Zohaib Khan, Saima Afaq

**Affiliations:** 1 Institute of Public Health & Social Sciences, Khyber Medical University, Peshawar, Khyber Pakhtunkhwa, 25000, Pakistan; 2 Office of Research, Innovation & Commercialization (ORIC), Khyber Medical University, Peshawar, Khyber Pakhtunkhwa, 25000, Pakistan; 3 Department of Health Sciences, University of York, York, England, YO10, UK

**Keywords:** Implementation science, innovation, innovation characteristics, intervention characteristics, integrated care

## Abstract

**Background:**

Tuberculosis with co-morbid diabetes mellitus presents a substantial public health challenge, necessitating immediate and coordinated interventions. Such interventions should be sustainable and have a high acceptance rate in real-world settings. Applying the principles of implementation science is essential to enhance the existing system. This research study aims to assess the influence of innovation characteristics on the implementation of an Optimised TB-diabetes integrated care package.

**Objectives:**

The main objectives of this study are to explore the perceptions and experiences of intervention developers; to understand the experiences of health professionals and patients regarding the Opt TBD integrated care package; assess the content validity of the innovation characteristics instrument; and evaluate the influence of intervention characteristics on the implementation process of the Opt TBD integrated care package.

**Methods:**

The study will be conducted at thirteen selected TB healthcare facilities across Khyber Pakhtunkhwa and Punjab over a period of 18 months, in three phases. Evaluation will involve qualitative exploration of intervention development, followed by feasibility testing, and finally the definitive implementation of the intervention. The participants will include key stakeholders: intervention developers, TB health professionals, and patients.

**Conclusion:**

This study will generate critical insights for the Opt TBD integrated care package, focusing on enhancing contextual relevance and improving adoption rates in practical, real-world settings. Specifically, this research study seeks to identify key implementation challenges, evaluate the fidelity of the intervention, and validate the causal relationships between the characteristics of the innovation and its implementation success.

**Abbreviations**
CFIR: Consolidated Framework for Implementation Research
CIs: Confidence Intervals
DC: Data Collection
DM: Diabetes Mellitus
DOTs: Directly Observed Treatment Short-course
GCP: Good Clinical Practice
HBA1c: Hemoglobin A1c
HEC: Higher Education Commission
KMU: Khyber Medical University
LMICs: Low- and Middle-Income Countries
MMAT: Mixed Methods Appraisal Tool
MO: Medical Officer
Opt TBD: Optimized Tuberculosis Diabetes
ORIC: Office of Research, Innovation, and Commercialization
QUAL: Qualitative
QUANT: Quantitative
RBG: Random Blood Glucose
TB: Tuberculosis
The Union: International Union Against Tuberculosis and Lung Disease
WHO: World Health Organization

## Insights and contributions of this research study

• This study protocol goes beyond a standard research design; owing to the limited guidance available in the literature, this protocol provides a novel perspective on implementation science, which is particularly beneficial for researchers focusing on innovation characteristics.
• By addressing existing knowledge gaps, this study enhances our comprehension of the role that innovation characteristics play in the dynamics of the implementation process, and their contribution to intervention’s success or failure and serves as a crucial resource for future researchers.
• The validated innovation characteristics tool will be submitted for inclusion in the CFIR Measures Gallery to facilitate its dissemination and use in implementation research.
• Furthermore, it contributes to refining and validating the CFIR model in the context of healthcare innovation.

## Background

The dual burden of tuberculosis (TB) and diabetes mellitus (DM) presents a pressing public health challenge, requiring immediate and well-coordinated interventions. ^1^ The risk of tuberculosis (TB) is increased three-fold in Diabetes Mellitus (DM) patients, and the likelihood of poor TB treatment outcomes is doubled. ^2, 3^ In 2021, the worldwide prevalence of diabetes among individuals aged 20–79 years was anticipated at 10.5% (536.6 million people), and this figure is predicted to rise to 12.2% (783.2 million) by 2045. ^4^ The International Diabetes Federation reported an alarmingly high figure in 2022, stating that 26.7% of adults in Pakistan were affected by diabetes; approximately 33 million cases were reported which continue to rise every year. ^5^ The World Health Organization (WHO) recommends collaborative care for TB patients with comorbid diabetes in its “Collaborative Framework for Care and Control of TB and Diabetes” *.* Based on this framework, the “International Union Against Tuberculosis and Lung Disease (The Union)”, developed a guide that incorporates evidence from published literature, expert opinions, and practical experiences to offer essential information for the applied and comprehensive management of TB patients with comorbid diabetes. ^6, 7^

With the knowing prevalence of TB and diabetes in Pakistan, an integrated approach to care is significantly required to ensure efficient resource utilization and improve health outcomes for both diseases. Therefore, an intervention is being delivered under the following research project: *“Implementation strategies for providing optimised tuberculosis and diabetes integrated care in LMICs: POTENTIAL”.* ^8^ The intervention, such as the Optimised TB Diabetes integrated care package (Opt-TBD), will combine the management of tuberculosis and diabetes into a single, coordinated plan. This intervention consists of two main components:

1. The **Opt TBD pathway** consists of Diabetes screening of TB patients through a Random Blood Glucose (RBG) test, confirmation of diabetes diagnosis with HBA1c, followed by management of both TB and Diabetes. MO/DOTs will review the treatment during routine follow-ups, and as required patients will be referred to specialised care. Follow-ups at the 3 ^rd^ and 6 ^th^ months for Opt-TBD will involve counseling and blood glucose measurements.
2. **Opt-TBD counseling** consists of sessions on Tuberculosis, Prediabetes and Diabetes, Healthy Lifestyle and Smoking cessation at 3 time points (Months 0, 3 & 6).

Thus, the methodologies of our research study will assess the Optimised TB Diabetes integrated care package (Opt-TBD), throughout its implementation process. Various earlier studies did focus on integrating TB and Diabetes co-management in Pakistan, however faced several limitations. These studies mostly focused on the feasibility of bidirectional screening ^9^ or determining the prevalence ^10^ of diabetes among TB patients. The implementation of integrated care has numerous challenges, such as the absence of medical equipment; inadequate skills and knowledge training; incomplete records and report systems; uncooperative integration; limited feedback; lack of referral systems; a shortage of supporting agencies; a lack of trained health worker force and focal persons; increased workload; and a lack of awareness. ^11^ For any intervention to be successful, the contextual relevance, which ensures the sustainability of the intervention, is crucial. ^12^

Implementation efforts, despite well-developed execution plans, often fail due to the underestimation of the contextual factors that hinder success in real-world settings. ^12^ One of the most crucial steps in implementing an intervention is understanding its characteristics and the challenges encountered during its implementation process. ^12^ The Consolidated Framework for Implementation Research (CFIR) serves as one of the most widely used determinant frameworks to evaluate and address these challenges effectively. ^12^ CFIR has five main domains: innovation domain, outer setting domain, inner setting domain, individuals domain and implementation process domain. The first domain ‘Innovation Characteristics’ focuses on the development of the intervention by exploring the *relative advantage, complexity, trialability, intervention source, design quality and packaging, evidence strength and quality, adaptability, and cost* of the intervention. ^13^ These aforementioned eight characteristics are explicit to each intervention and its context and are essential for successful implementation. ^13^

Lyon and Bruns in 2019, ^14^ highlighted “Innovation Characteristics” as a neglected area within the field of implementation science. Similarly, Thomas Engell in 2021, stated in a study that many previous implementation theories and frameworks articulated “Innovation Characteristics” as important determinants of successful implementation in healthcare settings but few studies have explored such characteristics in-depth. ^15^ The research gap exists in the literature indicating the limited evidence from Pakistan on the characteristics of innovations targeting the co-management of TB and diabetes along the implementation pathway. This research aims to address this gap by providing valuable insights into how the innovation characteristics of the Optimised TB Diabetes integrated care package (Opt-TBD), influence the stages of innovation implementation and ultimately determine their success or failure. The specific objectives of the study are as follows:

1. To explore the perceptions of developers of the OPT-TBD care package and relevant stakeholders regarding the design, development, and implementation process, with a specific focus on the innovation characteristics of CFIR.
2. To understand the trialability and complexity of the OPT-TBD care package and to generate insights for refining the intervention based on the innovation characteristics for definitive implementation.
3. To establish the content validity of the measurement instrument for innovation characteristics through Lawshe’s Content Validity Ratio (CVR) method.
4. To assess the influence of innovation characteristics on the implementation process of the OPT-TBD care package.

## Methods

**Figure 1.**
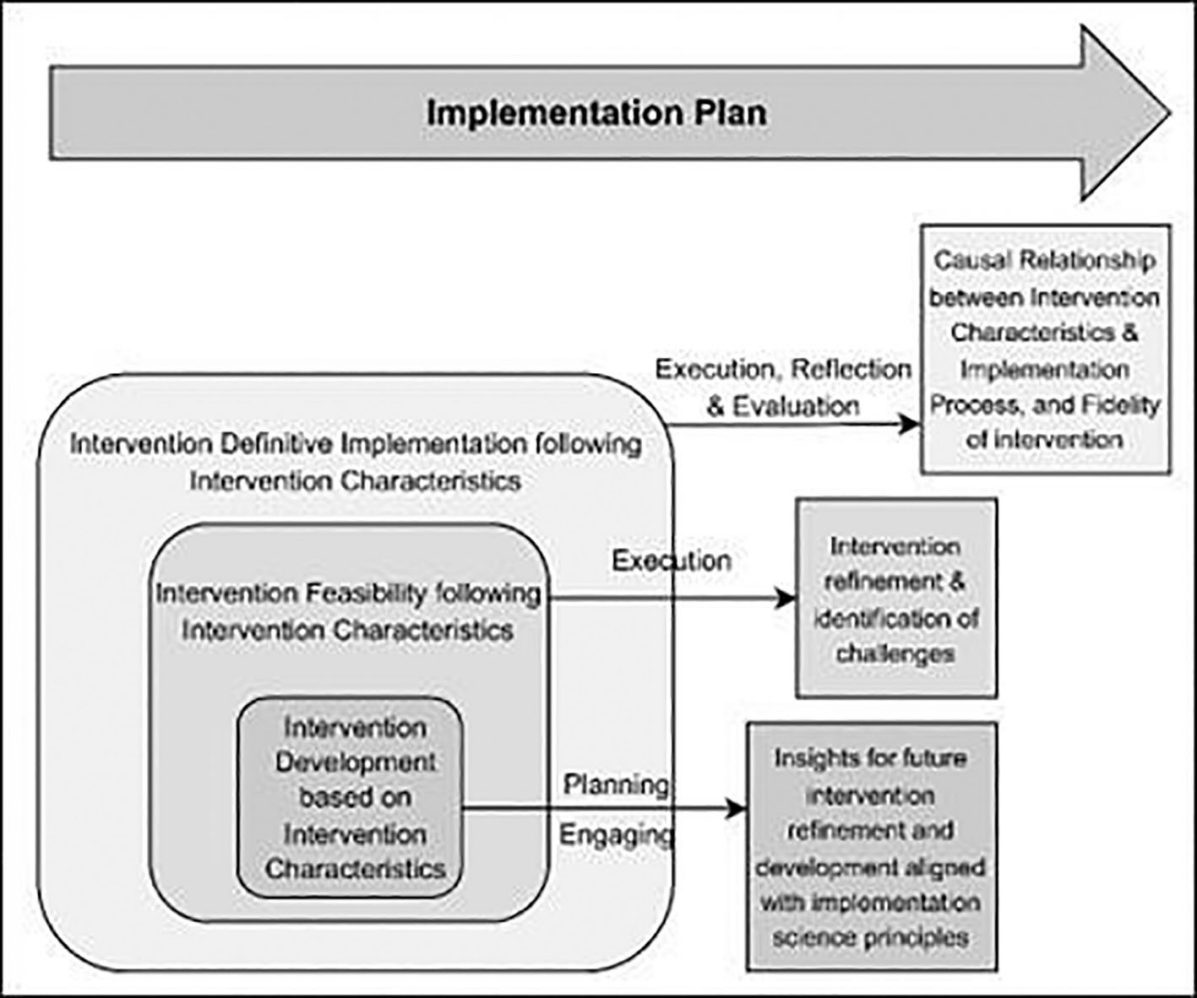
Conceptual Framework based on the CFIR model for evaluating the Optimised TB Diabetes integrated care package (Opt-TBD): illustrating the association between intervention characteristics and implementation process.

The Consolidated Framework for Implementation Research (CFIR) provides a comprehensive and systematic approach to understanding, analysing, and improving the implementation of health interventions. ^13^ This study will be theoretically underpinned by the CFIR model, with a specific focus on the domain of innovation characteristics, accompanied by guidance from the process domain. ^16^ The conceptual framework of this research study is shown in Figure 1, below:

### Study settings and design

The Opt-TBD care package intervention is being implemented at thirteen purposefully selected TB care facilities across the provinces of Khyber Pakhtunkhwa and Punjab in Pakistan, over a period of 18 months. These facilities include a combination of primary, secondary, and tertiary care settings to ensure that the intervention can be implemented and evaluated across different healthcare levels.

A fully integrated convergent parallel ^17^ mixed-method design is employed, in which quantitative and qualitative data are collected and analyzed simultaneously. This approach enhances the depth and validity of the research findings by enabling the triangulation of results, thereby providing a more comprehensive and nuanced understanding of both the implementation process and contextual influences. ^18^

### Data collection method & tools

Qualitative and quantitative approaches will be employed to gather insights from the Opt TBD developers, TB patients with diabetes, DOTS facilitators, and medical officers. Table 1 below represents the scheduled timing of all data collection tools during the Opt TBD care package implementation. Tables 2 and 3 mentioned below, outline these methods and their respective participants. The qualitative data collection instruments, comprising three topic guides, were independently developed based on the CFIR model to ensure theoretical grounding and contextual relevance. The guides underwent critical review by a multidisciplinary panel, including a qualitative research expert, an implementation scientist, and a public health researcher, to enhance content validity and methodological rigor. In parallel, a quantitative survey instrument designed to assess perceived innovation characteristics was developed in alignment with Objective 3 of the study. This self-developed tool is conceptually grounded in the Consolidated Framework for Implementation Research (CFIR). An extensive review informed item generation from existing literature and was subsequently adapted and contextualized to reflect the components of the OPT-TBD care package. The instrument will undergo expert validation prior to its application in Objective 4 to ensure content validity, relevance, and clarity. The implementation outcomes questionnaire (IOQ), which is freely available online, will be utilized in the context of the OPT TBD care package to assess the progress and effectiveness of the implementation efforts within this study. This tool will be piloted first before its full application in Objective 4 along with the innovation characteristics instrument.

**Table 1.** Schedule of the data collection methods at different timepoints.

	Study measures	Time point		
		0 month	3 ^rd^ month	6 ^th^ month
**Qualitative Data**	In-Depth Interviews (IDIs) (Intervention Developers)	✓	✗	✗
	Semi Structure Interviews (SSI) (TB & DM patients)	✗	✗	✓
	Semi Structure Interviews (SSI) (MOs & DOTS facilitators)	✗	✗	✓
**Quantitative Data**	Expert content validity of the measurement instrument for innovation characteristics- Round 1	✓	✗	✗
	Expert content validity of the measurement instrument for innovation characteristics- Round 2	✗	✓	✗
	Innovation Characteristics Survey	✗	✗	✓
	Implementation Outcomes Questionnaire (IOQ)	✗	✗	✓

**Table 2.** Overview of innovation characteristics, data collection methods, and corresponding participant groups.

Sr. No	Innovation characteristics	Brief description	Measuring tool		Group (Intervention developers, TB health workers and TB-DM patients)	Frequency
			Qualitative data	Quantitative data		
**1**	**Innovation Source**	The group of stakeholders involved in the development and visible sponsorship of the OPT TBD care package is reputable, credible, and trustworthy.	Qualitative interviews		Intervention Developers	1
**2**	**Evidence-Based Innovation**	The OPT TBD care package has robust evidence supporting its effectiveness.	Qualitative interviews	Innovation Characteristics Survey	Intervention developers, TB health workers and TB-DM patients	2
**3**	**Relative Advantage**	The OPT TBD care package is better than other previously available interventions or current practice.	Qualitative interviews	Innovation Characteristics Survey	Intervention developers, TB health workers and TB-DM patients	2
**4**	**Adaptability**	The OPT TBD care package can be modified, tailored, or refined to fit local context or needs.	Qualitative interviews	Innovation Characteristics Survey	Intervention developers, TB health workers and TB-DM patients	2
**5**	**Trialability**	The OPT TBD care package can be tested or piloted on a small scale.	Qualitative interviews	Innovation Characteristics Survey	Intervention developers, TB health workers and TB-DM patients	2
**6**	**Complexity**	The OPT TBD care package is complicated, which may be reflected by its scope and/or the nature and number of connections and steps.	Qualitative interviews	Innovation Characteristics Survey	Intervention developers, TB health workers and TB-DM patients	3
**7**	**Design and packaging**	The OPT TBD care package is well designed and packaged, including how it is assembled, bundled, and presented.	Qualitative interviews		Intervention developers	2
**8**	**Cost**	The OPT TBD care package purchase and operating costs are affordable.	Qualitative interviews		Intervention developers	1

**Table 3.** Summary of process indicators, data collection tools, frequency, and target participant groups.

S. No	Process	Measuring indicators	Measuring tools (Quantitative survey)	Group	Frequency
**1**	Planning	Appropriateness	Implementation Outcomes Questionnaire, 2021	TB health workers and TB-DM patients	1
**2**	Engaging	Adoption	Implementation Outcomes Questionnaire, 2021	TB health workers and TB-DM patients	1
		Acceptability			
**3**	Execution	Feasibility	Implementation Outcomes Questionnaire, 2021	TB health workers and TB-DM patients	1
		Fidelity	Checklist ( Framework Implementation fidelity)		
**4**	Reflection & Evaluation	Penetration	Implementation Outcomes Questionnaire, 2021	TB health workers and TB-DM patients	1

We will first detail the qualitative methods for objectives one, two, and four of the study. Next, we will describe the quantitative methods for objectives three and four. For all four objectives, the data collection will be based on the constructs of the first domain, which represent the innovation characteristics of the CFIR model. ^16, 19^ We have adhered to the Medical Research Council guidance on developing and evaluating complex interventions, emphasising the significance of engaging all relevant stakeholders. ^20^ The first author will undertake primary data collection. To ensure efficient data collection across multiple study sites and adherence to project timelines, two trained research assistants will be engaged if additional logistical or operational support is needed.

### Qualitative data

Qualitative data collection serves as the foundational component of this study, enabling an in-depth exploration of implementation experiences across three distinct stages. Guided by the CFIR framework, the inquiry specifically focused on the domain of ‘Innovation Characteristics’ to examine how perceived attributes of the OPT TBD care package influenced its implementation across the TB settings.

### Data collection tool ( Table 2)

Topic guides for the three qualitative data collection stages will be systematically developed using the eight core constructs of the ‘Innovation Characteristics’ domain from the Consolidated Framework for Implementation Research (CFIR). ^16, 19^ These constructs will serve as the analytical lens guiding data collection. In Stage 1, which involves interviews with intervention developers, the topic guide will encompass all eight constructs: *intervention source, evidence strength and quality, adaptability, relative advantage, trialability, complexity, design and packaging, and cost.* Stage 2 will focus specifically on *trialability and complexity*, explored through interviews to identify necessary adaptations and challenges before definitive implementation. In the final stage 4 of qualitative data collection, the topic guide will be designed to assess *evidence strength and quality, adaptability, relative advantages, complexity, design and packaging*, drawing on the lived experiences of implementers and end-users to evaluate the intervention’s perceived value and sustainability.

These guides will be reviewed and finalized with input from a qualitative expert, a public health expert, and implementation science experts. They will serve as a structured framework to capture in-depth insight into participants’ experiences, perceptions of the intervention, the contextual and operational factors influencing its delivery, as well as barriers and facilitators to scaling up. A total of three distinct topic guides will be developed, tailored to intervention developers, DOTS facilitators, Medical Officers (MOs) and TB diabetes patients.

### Stage 1: Source, evidence-based innovation, adaptability, relative advantage, trialability, complexity, design & packaging and cost

This is the first phase of a study which aimed to explore intervention developers’ perceptions and experiences regarding the development of OPT TBD care packages based on the innovation characteristics of CFIR.

### Data collection, participants & sampling

A purposive maximum variation sampling technique will be employed to identify participants. We will select participants involved in the intervention’s development, ensuring representation by choosing one participant from each group at every stage, thereby enhancing the transferability and richness of findings. Selection will be based on the following criteria: the participant’s experience in intervention development, their role within the development team (e.g., lead, co-investigator, researcher), their professional background (such as clinician, public health expert), service users such as patients, and the type and setting of the intervention. ^21^ A target of approximately 10 participants will be recruited, aiming to include at least one representative from each identified stakeholder group.

The data collection will be initiated through formal email invitations, which will include a participant information sheet that explicitly outlines the study’s purpose, confidentiality measures, audio recording procedures, and the voluntary nature of participation. Written consent form will be taken from all the participants of the study. Non-responders will receive two reminder emails over a two-week interval.

Interviews will be conducted via Zoom for remote participants and in person where feasible, depending on participants’ locations and availability. All interviews will follow a semi-structured format guided by a piloted interview guide mapped to relevant CFIR innovation constructs. Interviews will be audio recorded, transcribed verbatim, and supplemented by brief field notes. Demographic data will be systematically collected using a standardised form.

Data collection will span approximately 8 to 12 weeks, and all procedures will be documented in a study log.

### Stage 2: Trialability & complexity

The trialability of an intervention is crucial, because it helps identify the operational challenges, contextual fit and complexity of its components at a small-scale level. Therefore, this stage of the study is conducted to understand the trialability and complexity of the OPT-TBD care package. Insights from this phase will guide the refinement of the intervention to enhance its adaptability for broader implementation, in line with CFIR innovation characteristics.

### Data collection, participants and sampling

This stage of study involves data collection from two key participant groups: 1) Intervention deliverers such as TB health workers (DOTS facilitators and medical officers) and 2) Intervention recipients, consisting of TB patients diagnosed with diabetes and those who will receive OPT TBD care package.

The OPT TBD care package is initially implemented at small level only at four sites: three in KP and one in Punjab. We will conduct qualitative interviews with four DOTS facilitators, four MOs, and eight patients, ensuring a balanced representation of gender and settings and data saturation. The intervention will be delivered in three stages at the TB facility (at 0, 3, and 6 months). Therefore, qualitative semi-structured interviews will be held with the intervention deliverers (DOTs & MOs) and intervention recipients (patients) at the 6-month time point, after the complete delivery of the intervention. All participants will complete demographic forms, and there will be an information sheet containing all the details about the research study. Before starting the interview, participants will be asked to sign consent forms, and their interviews will be audio recorded. Data collection will span approximately 8–32 weeks, and all procedures will be documented in a study log.

### Stage 4: Evidence-based innovation, adaptability, relative advantage, complexity, design and packaging

In the definitive implementation phase, collecting qualitative data is critical to gaining an in-depth understanding of the challenges faced by end users during the real-world delivery of the OPT-TBD care package. Specifically, this phase aims to explore perceptions related to the adaptability and relative advantage of the intervention based on the first hand experiences of both providers and recipients. To complement and contextualise the quantitative findings, a series of semi-structured interviews will be conducted at the six-month point, following the complete delivery of the care package across all implementation sites.

### Data collection, participants and sampling

The definitive implementation will take place across 13 designated TB care facilities. To ensure site-level representation, one patient and one DOTS facilitator will be purposively selected from each site, resulting in a total of 26 interviews, ensuring the data saturation. These participants will include intervention deliverers (DOTS facilitators) and intervention recipients (patients with TB and diabetes).

Before the interviews, participants will complete a demographic information form and receive a detailed participant information sheet outlining the study’s purpose, procedures, risks, and benefits. Written informed consent will be obtained from all participants, including consent for audio recording. Face-to-face interviews will be conducted, digitally recorded, transcribed verbatim, and anonymized. Data collection is expected to occur over 8 to 32 weeks, depending on site readiness and participant availability.

### Quantitative data

**Objective 3: Innovation characteristics tool validation**

To ensure methodological rigour and enhance the reproducibility of the innovation characteristics tool, a structured, two-phase process will be implemented to establish its content validity. The development of the tool will be informed by a comprehensive literature review of key implementation science frameworks, particularly the Consolidated Framework for Implementation Research (CFIR), which outlines core constructs such as evidence-based innovation, adaptability, trialability, relative advantage, and complexity. This review will guide the conceptual definition and operationalization of each construct within the specific context of the OPT-TBD care package. Based on this foundational work, an initial pool of items will be generated to capture the theoretical breadth of the selected constructs. The rationale for undertaking a formal content validity assessment is to ensure that the instrument adequately represents the intended construct domains and demonstrates contextual relevance across diverse healthcare implementation settings.

### Data collection, participants and sampling

Content validity will be assessed using Lawshe’s Content Validity Ratio (CVR) methodology. A panel of 10–14 subject matter experts, including implementation researchers, public health professionals, TB/diabetes clinicians, and medical health professions education specialists, will be purposively selected based on their domain expertise. Experts will be contacted via email and invited to participate in two rounds of item evaluation using structured questionnaires administered through Google Forms.

In each round, experts will independently evaluate the clarity, relevance, and representativeness of each item using a 4-point ordinal scale (i.e., very clear, clear, somewhat clear, or not at all clear). CVR scores will be calculated for each item based on expert consensus, with items retained, revised, or discarded according to Lawshe’s critical value thresholds. Furthermore, the Item- and Content Validity Ratio (I-CVI and CVR) will be computed to further inform item-level decision-making. The tool will be iteratively refined after each round, based on both quantitative indices and qualitative expert feedback in the suggestion column. The entire expert validation and data collection process will be completed within a two-month timeframe. All procedures will be documented in a study log to ensure transparency. The end goal is to produce a psychometrically sound instrument capable of reliably measuring the innovation characteristics relevant to implementation outcomes in real-world health systems and to establish a way forward to collect data on objective 4 of this study.

**Objective 4: Association between innovation characteristics & implementation outcomes**

To evaluate how specific innovation characteristics influence the implementation process, a structured mixed-methods approach will be adopted during the definitive implementation phase of the refined OPT-TBD intervention. Quantitative and qualitative data will be collected concurrently from TB health workers and patients receiving the OPT TBD care package across all 13 implementation sites. Qualitative data have already been explained in the previous section, while the quantitative section is stated below.

### Data collection tools ( Tables 2 & 3)

The measurement of innovation characteristics, including evidence-based innovation, adaptability, trialability, relative advantage, and complexity, will rely on an instrument developed and content-validated under Objective 3. Following pilot testing on 10% of the sample for clarity and feasibility, this tool will be deployed during the definitive implementation phase to collect data from both TB and diabetes patients, as well as TB health workers (DOTS facilitators and medical officers) involved in delivering the OPT-TBD care package.

On the other hand, implementation outcomes will be assessed using the implementation outcome questionnaire, ^22^ which captures core constructs such as acceptability, adoption, appropriateness, and feasibility. These outcomes will provide quantitative insights into the perceived effectiveness and operational integration of the care package.

The fidelity of implementation will be assessed using a structured checklist developed under the POTENTIAL project, grounded in the conceptual framework for implementation fidelity. ^23^ This checklist will guide both observational assessments and audio-recorded fidelity monitoring. Because this research is nested within the broader POTENTIAL project, the same fidelity data will be utilised to ensure consistency, comparability, and traceability of implementation fidelity measures across sites and phases.

### Data collection, participants and sampling

A census-based sampling strategy will be used to include all eligible participants. Quantitative data will be gathered from the entire cohort of almost 250 patients, as well as from all DOTS facilitators and MOs involved in delivering the intervention. Approximately 250 patients with confirmed co-diagnoses of tuberculosis and diabetes are expected to be recruited under the POTENTIAL project and will be provided with the OPT-TBD integrated care package as part of the planned intervention delivery. ^8^ The census approach ^24^ is scientifically justified given the relatively small, accessible population size, allowing for maximal coverage, elimination of sampling bias, and detailed assessment of inter-individual variability in implementation responses. All participants will be informed through a detailed study information sheet and written informed consent will be taken before completing hard-copy questionnaires.

Fidelity to the intervention protocol will be rigorously assessed. The OPT-TBD intervention will be delivered in three stages (baseline, 3 months, and 6 months). Audio recordings of intervention delivery sessions will be collected at each stage from five districts, with two audio recordings per district (total n = 10). Three independent reviewers will score each recording on the checklist, with discrepancies resolved through consensus to ensure inter-rater reliability. To further validate fidelity, data from the POTENTIAL project ^8^ will be leveraged.

All data collection activities, including survey administration, audio recordings, and fidelity assessments, will be completed within a six-month timeline, with detailed documentation of procedures maintained in a central study log to ensure reproducibility and auditability.

### Data confidentiality

The confidentiality of all the personal data will be securely maintained throughout the study’s duration and afterwards. Only essential personal information will be collected and linked to participant IDs on a demographic sheet. The correspondence details will be deleted once they are no longer required. All data will be anonymised by giving participant IDs and will be stored at Khyber Medical University’s assigned locker to the first author. Audio recordings of all three phases of the interviews will be captured via an encrypted audio recorder.

### Data analysis plan

In this research study, a fully integrated convergent parallel mixed method design (QUAL (dc) QUAL (dc)QUANT (dc) + QUAL (dc) Analysis Integration of data) ^17^ will be used to assess the influence of innovation characteristics on the implementation process of an optimised TBD care package. The parallel collection and analysis of quantitative and qualitative data for a better understanding of the causal relationship between innovation characteristics and the implementation process will be performed. Using both methods help confirm that the results converge to the same conclusion.

### Qualitative analysis

Audio-recorded interviews, from all three stages, will be transcribed word-for-word to capture every detail accurately. The data from these interviews will be analysed via thematic framework analysis. This approach combines both inductive and deductive methods. The process begins with familiarising ourselves with the data and developing a framework. Following this, we proceed with indexing the data, organising it into charts, and finally interpreting the findings. Microsoft Excel will be utilised to assist with these stages of data management and analysis.

### Quantitative analysis

**Objective 3: Content validity analysis**

To assess the content validity of the innovation characteristics measurement tool, a systematic analysis will be conducted using Microsoft Excel. Two rounds of expert review will be carried out, involving a panel of subject matter experts who will evaluate each item for relevance, clarity, and representativeness. The Item-Level Content Validity Index (I-CVI) and the Content Validity Ratio (CVR) will be computed for each item following established guidelines. An I-CVI threshold of ≥0.78. ^25^ will be used to indicate excellent content validity, as recommended for tools evaluated by more than six experts. For CVR, the minimum acceptable value was determined based on Lawshe’s critical values; for example, a CVR threshold of ≥0.49 was applied when 15 experts participated. ^25^ Items falling below these thresholds were either revised for improved clarity and alignment with construct definitions or removed from the instrument. The analysis will be conducted in Excel using binary coding (1 = essential, 0 = not essential) for CVR and relevance ratings for I-CVI, ensuring transparency and reproducibility of scoring. This rigorous process ensured that the final tool demonstrated high content validity for use in evaluating innovation characteristics in healthcare implementation settings.

**Objective 4:**

The data will be collected during stage 4, coinciding with the definitive implementation of the intervention’s Opt TBD integrated care package.

*Variables:*

*Independent variables:* The independent variables in this study include, *Evidence-based innnovation, Relative Advantage, Adaptability, Trialability and Complexity.* Each of these variables is measured consistently across stage 4, ensuring a standardised approach to data collection.

*Dependent variables:* The process domain of CFIR has a total of four constructs: planning, engaging, execution, and reflection & evaluation. In this research study, we will use proxy measures to assess these constructs by utilising the Implementation Outcomes Questionnaire (2021). ^30^ According to this questionnaire and aligned with the objectives of our study using CFIR, the measure for planning will be appropriate, for engaging it will be adoption and acceptability, for execution it will be feasibility and fidelity (with fidelity being measured separately through a checklist), and the measure for evaluation will be penetration.

*Analysis Plan:* Initially, percentages, means, and standard deviations will provide a foundational understanding of the data distribution. Following this, inference analysis will be employed, utilising ANOVA for mean comparison and Post Hoc tests to further differentiate between the variables. The significance of associations will be determined using the P-value. Finally, regression analysis will be performed, employing Multinomial Logistic Regression, to assess the associations between exposure and outcomes, adjusting for a set of covariates. All the statistical data will be analysed using the software STATA version 14.0.

**Fidelity:** The Fidelity index scale will be assessed as frequencies and percentages using 95% of confidence intervals (CIs), aimed at the delivery of innovation in terms of a *3-point Likert scale that is 0-Missed, 1- Partial, and 2 complete.* A higher Likert value indicates that innovation is being delivered according to the specifications as designed in terms of adherence or adaptability, quality, and quantity.

### Integration of data

To ensure optimal results and achieve convergent validity between the quantitative and qualitative methods, data integration will be employed. ^26^ The data from phase four will be integrated with findings from phase one, which involved qualitative interviews with intervention developers. This integration of data aims to provide a comprehensive understanding of the intervention’s success. The results from phase three will validate the adherence to the innovation domain during the intervention’s development, suggesting that it exhibits lower complexity and higher acceptability and adoption rates among patients. Furthermore, these findings will confirm that interventions designed in alignment with the eight constructs of the innovation domain are more likely to achieve higher adoption and penetration rates within the healthcare system.

## Discussion

Our research employs a mixed-methods approach to thoroughly explore the role of implementation science in the development of interventions, their feasibility, and the causal relationship between innovation characteristics and the implementation process of the OPT-TBD integrated care package. Carefully studying the implementation process and addressing any challenges encountered is vital for the success of these interventions.

According to the literature, complex interventions involve multiple steps and various components. ^27^ The process of intervention development is a crucial factor in the effective implementation of the intervention, ^27^ and the success of this implementation relies on whether the intervention is evidence-based and delivered as intended. ^20^ Furthermore, participants must be willing to implement it, and the acceptability and adoption rate of the intervention should be high. ^20^ Information is scarce regarding the steps used in developing interventions implemented in real-world settings. ^28^ In the initial phase of this research study, qualitative data will be collected to explore the processes involved in designing and developing the OPT-TBD integrated care package. The findings from this phase aim to inform a systematic framework for future health intervention development, facilitating the design of contextually relevant and acceptable interventions with a higher likelihood of successful implementation.

A research study highlighted that most of the global disease burden is concentrated in low and middle-income countries (LMICs), where many effective interventions are recommended for implementation and scaling up across health systems. ^29^ However, these interventions are typically not adopted. Thus, to optimise and sustain the implementation of such interventions in LMICs, locally driven implementation research is essential. ^29^ Implementation research in LMICs presents unique opportunities to identify key barriers to the adoption, scaling up, and sustainability of evidence-based interventions in these settings. ^30^ Literature suggests that collaborative research efforts involving patients, communities, non-governmental organizations, academia, and government ministries of health and finance can help develop sustainable, context-specific solutions tailored to local needs. ^29, 30^ The insights from our study will identify challenges encountered during implementation, offering valuable guidance for addressing barriers and enhancing the contextual relevance and acceptability of the care package. This study will also confirm that using the principles of implementation science can enhance the implementation and sustainability of intervention. The recent research indicates that there is a causal relationship between innovation characteristics and the implementation process. ^12^ For this reason, a convergent mixed-method study will be utilised to establish evidence, gathering both quantitative and qualitative data to determine the associations between innovation characteristics and the implementation process.

The study’s strength lies in its mixed-methods design, which integrates qualitative and quantitative data to provide a holistic understanding of the implementation process, thereby enhancing the validity of the findings. Grounded in the CFIR model, it offers a structured evaluation of innovation characteristics, rendering the results robust and applicable for refining strategies. Engaging diverse stakeholders ensures a range of perspectives while contributing valuable evidence-based recommendations for optimizing integrated care. However, there are some limitations, including limited generalizability due to its focus on selected TB care facilities in Pakistan, which may not represent other settings. Resource constraints and variations in healthcare infrastructure may impact the feasibility and scalability of the Opt-TBD package in different regions.

### Dissemination

The findings from this study will contribute to the primary author’s doctoral dissertation and will be presented during their thesis defence at the university. Additionally, a summary report of the results will be compiled and shared with the TB programs in KP and Punjab, as well as with study participants upon request. The results will also be submitted for consideration in an international peer-reviewed journal.

### Study status

OPT TBD care package is in definitive implementation stage at the moment.

## Ethical consideration

The study has received a favourable ethics opinion from the Advanced Study Research Board (ASRB) and the Institute of Public Health & Social Sciences, Ethics Committee of Khyber Medical University, Pakistan, under reference no. KMU/IPHSS/Ethics/2024/DH/0195. Letters of permission and support have been obtained from the TB programs of both Khyber Pakhtunkhwa and Punjab Provinces. Written Informed consent will be obtained from all individual participants included in the study, in accordance with ethical standards. All data will be handled according to the Good Clinical Practice (GCP) guidelines.

## Data Availability

No data associated with this article. **Figshare:** Supplementary Documents- Assessing the Influence of Innovation Characteristics on the Implementation Process of an Optimised Tuberculosis Diabetes Integrated Care Package (Opt TBD) -A Mixed Method Study Protocol, https://doi.org/10.6084/m9.figshare.29064476.v2 ^31^ This project contains the following underlying data:
• Consent form & Information Sheet • Participant Demographic forms • Qualitative Topic Guides • Quantitative Questionaires Data are available under the terms of the Creative Commons Attribution 4.0 International license (CC-BY 4.0).
