# Supplementary material for: Assessing the Influence of Innovation Characteristics on the Implementation Process of an Optimised Tuberculosis Diabetes Integrated Care Package (Opt TBD) -A Mixed Method Study Protocol
Source: F1000Res. 2026 Jan 9;14:599. Originally published 2025 Jun 18. [Version 3] doi: 10.12688/f1000research.165171.3 (PMC12856243; doi:10.12688/f1000research.165171.3)
Supplement: Peer review history

# Reviewer report: Reviewer response for version 3

**DOI:** 10.5256/f1000research.194708.r448975

**Published:** 2026-01-29

**Authors:** Falak Pahwa

**Affiliations:** 1 The University of Chicago (Ringgold ID: 2462), Chicago, Illinois, USA

The authors have adequately addressed the concerns raised in the previous review. 

 These modifications collectively improve methodological transparency, clarity, and overall scientific rigor. No further revisions are needed at this time.

Is the study design appropriate for the research question?

Yes

Is the rationale for, and objectives of, the study clearly described?

Yes

Are sufficient details of the methods provided to allow replication by others?

Yes

Are the datasets clearly presented in a useable and accessible format?

Yes

Reviewer Expertise:

TB immunology

I confirm that I have read this submission and believe that I have an appropriate level of expertise to confirm that it is of an acceptable scientific standard.

# Reviewer report: Reviewer response for version 2

**DOI:** 10.5256/f1000research.190451.r428585

**Published:** 2025-11-20

**Authors:** Falak Pahwa

**Affiliations:** 1 The University of Chicago (Ringgold ID: 2462), Chicago, Illinois, USA

This study protocol by Khalid F et al is written very extensively on the perturbing disease tuberculosis (TB) along with an emerging lifestyle disease- diabetes (DB). Interventions to have a care for both TB-DB cases becomes very important in TB endemic regions like Pakistan which is having an increasing emergence in DB cases. All the questionnaires enlisted are detailed and pave a great discussion to have OPT-TBD package implemented along with its innovation characteristics.

 However, there are some minor points, which needs to be addressed:

1. The authors do mention taking n=250, but a description of age and sex of the patients should be mentioned. TB and DB is more prevalent in males but females have a higher risk of developing complications.
2. In the patient questionnaire, it would be helpful if a disease history column is added below the co-morbid disease condition section.
3. The study will be carried out in provinces of Khyber Pakhtunkhwa and Punjab in Pakistan. It would be interesting to include Sindh since it has highest prevalence rates of DB.
4. There is repetition of text at many places, like abbreviations once introduced can be used as it is when used later in the text. And sometimes, many things gets lost in between the text. For example, the method sections talks about Objectives 3 and 4 directly and has no sub-heading for Objectives 1 and 2. Similarly, In the data collection, stage 3 seems to be missing from the text.

Is the study design appropriate for the research question?

Yes

Is the rationale for, and objectives of, the study clearly described?

Yes

Are sufficient details of the methods provided to allow replication by others?

Yes

Are the datasets clearly presented in a useable and accessible format?

Yes

Reviewer Expertise:

TB immunology

I confirm that I have read this submission and believe that I have an appropriate level of expertise to confirm that it is of an acceptable scientific standard, however I have significant reservations, as outlined above.

# Response

**Published:** 2026-01-01

**Authors:** Fatima Khalid

I sincerely thank the reviewer for their insightful and constructive comments. I

 have carefully addressed all points raised and revised the manuscript accordingly. Detailed responses are provided below.

 1) I have incorporated a demographic description of participants’ age and sex distribution in the data collection section. Track changes have been made, and the demographic form has also been uploaded along with the questionnaire, in which we will collect these details.

 2) I appreciate this recommendation. A co-morbid disease section has now been added to the patient questionnaire. The revised questionnaire has been updated accordingly and uploaded as supplementary file updated version.

 3) We thank the reviewer for this valuable observation. While we agree that inclusion of Sindh would strengthen generalizability, this was not feasible due to resource, logistical, and time constraints, as the study was conducted within the framework of a time-bound PhD program. We have now explicitly clarified this rationale in the Limitations section of the manuscript.

 4). Thank you for highlighting these structural and clarity issues; the manuscript has been revised to remove repetitive text, ensure consistent use of abbreviations after their first definition, reorganize the Methods section with clear alignment to Objectives 1, 2 and4, and explicitly include and describe Stage 3 in the Data Collection section.

# Reviewer report: Reviewer response for version 1

**DOI:** 10.5256/f1000research.181773.r402573

**Published:** 2025-09-04

**Authors:** Arohi Chauhan

**Affiliations:** 1 South Asian Institute for Health Promotion, Odisha, India

1. Clarity and Language
  - Some sections are wordy and repetitive, especially in the *Background* and *Methods*. Example: implementation challenges are listed twice in slightly different ways. Streamlining would improve readability.
  - Jargon-heavy phrasing (“convergent parallel QUAL (dc) QUAL (dc)!QUANT…”) could be simplified for broader audiences.
2. Sampling Strategy Justification
  - Census-based approach for quantitative phase is logical, but more detail is needed on potential recruitment barriers (e.g., attrition, refusal).
  - Clarify how “data saturation” will be operationalized in qualitative phases.
3. Data Analysis Details
  - Thematic framework analysis is appropriate, but further explanation on ensuring inter-coder reliability or reflexivity in coding would add transparency.
  - For quantitative analysis, multinomial regression is planned, but covariates are not clearly defined. Consider specifying which socio-demographic or clinical variables will be controlled.
4. Integration of Data
  - Integration strategy is mentioned but could be expanded with examples of how qualitative and quantitative findings will be *jointly displayed* (e.g., matrices, joint displays).
5. Limitations Section
  - While generalizability is acknowledged, other foreseeable limitations (e.g., reliance on self-report data, facility-level variation in readiness, challenges of fidelity monitoring in resource-limited settings) could be more explicitly discussed.
6. Dissemination
  - Dissemination is noted (PhD, reports, journal submission), but could highlight knowledge translation to policy and practice, especially how findings will be fed back into Pakistan’s TB and DM programs.

Is the study design appropriate for the research question?

Yes

Is the rationale for, and objectives of, the study clearly described?

Yes

Are sufficient details of the methods provided to allow replication by others?

Yes

Are the datasets clearly presented in a useable and accessible format?

Yes

Reviewer Expertise:

Tuberculosis, health system strengthening, multiple long-term conditions

I confirm that I have read this submission and believe that I have an appropriate level of expertise to confirm that it is of an acceptable scientific standard.

# Response

**Published:** 2025-10-28

**Authors:** Fatima Khalid

We thank the reviewer for the constructive comment.

 1. The Methods section was revised to remove repetitive descriptions of data collection and sampling procedures across Stages 1, 2, and 4. A unified subsection “Qualitative Data Collection Procedures” was added, and stage-specific paragraphs were rewritten to focus only on unique elements.

 We agree that the original notation used to describe the mixed-methods design could be difficult for readers who are not familiar with advanced mixed-methods conventions. We have revised the sentence to use plain language and now describe the mixed-methods approach as a “convergent mixed-methods design in which qualitative and quantitative data are collected and analyzed separately and integrated during interpretation.” This improves clarity and accessibility for broader audiences (Methods section, Mixed-Methods Design subsection).

 Additional detail has been added to clarify the sampling strategy. For the quantitative phase, the rationale for the census-based approach has been expanded to describe potential recruitment barriers (attrition, refusal, turnover) and planned mitigation strategies to support participation. For the qualitative phases, we have specified how data saturation will be operationalized and confirmed during analysis. These revisions have been incorporated in the Methods section (Sampling & Data Collection Procedures).

 2. We have now clarified the sampling strategy by adding detail on potential recruitment barriers and corresponding mitigation strategies for the census-based quantitative phase. Additionally, we have specified how data saturation will be operationalized and confirmed during qualitative analysis.

 3. Data Analysis Details: We have now added detail to clarify how inter-coder reliability and reflexivity will be ensured. Specifically, at least two researchers will independently code an initial subset of transcripts and reconcile coding decisions to develop a shared coding framework, with discrepancies resolved through discussion and senior review where needed. Reflexivity will be maintained through analytic memos and team debriefing sessions. This addition has been inserted into the Qualitative Analysis subsection of the Methods.

 We have now clarified the covariates to be included in the multinomial regression model. Specifically, age, gender, education level, province, facility type, and duration of TB-DM comorbidity will be included for patient-level analysis, while professional role, years of clinical experience, and prior training will be included for health worker data. These covariates were selected based on theoretical relevance and established determinants of implementation outcomes. This clarification has been added in the Quantitative Analysis section.

 4. We have expanded the Integration section to describe how qualitative and quantitative findings will be brought together using joint displays and matrices to support convergence and interpretation.

 5. We also strengthened the Limitations section to explicitly discuss self-report data, variation in facility readiness, and challenges related to implementation fidelity monitoring. 

 The Dissemination section has been revised to highlight knowledge translation activities, including engagement with the National TB Control Program and provincial health departments to support integration of findings into routine TB-DM programming. These revisions improve clarity, transparency, and relevance to policy and practice.
